# Genotoxicity and carcinogenicity of cobalt-, nickel- and copper-based nanoparticles

**DOI:** 10.3892/etm.2012.656

**Published:** 2012-08-07

**Authors:** RUTH MAGAYE, JINSHUN ZHAO, LINDA BOWMAN, MIN DING

**Affiliations:** 1 Department of Preventive Medicine of the Medical School, Zhejiang Provincial Key Laboratory of Pathological and Physiological Technology, Ningbo University, Ningbo, Zhejiang 315211, P.R. China;; 2 Pathology and Physiology Research Branch, Health Effects Laboratory Division, National Institute for Occupational Safety and Health, Morgantown, WV 26505, USA

**Keywords:** genotoxicity, carcinogenicity, nanoparticles, cobalt, nickel, copper

## Abstract

The nanotechnology industry has matured and expanded at a rapid pace in the last decade, leading to the research and development of nanomaterials with enormous potential. The largest source of these nanomaterials is the transitional metals. It has been revealed that numerous properties of these nano-sized elements are not present in their bulk states. The nano size of these particles means they are easily transported into biological systems, thus, raising the question of their effects on the susceptible systems. Although advances have been made and insights have been gained on the effect of transitional metals on susceptible biological systems, there still is much ground to be covered, particularly with respect to our knowledge on the genotoxic and carcinogenic effects. Therefore, this review intends to summarize the current knowledge on the genotoxic and carcinogenic potential of cobalt-, nickel- and copper-based nanoparticles indicated in *in vitro* and *in vivo* mammalian studies. In the present review, we briefly state the sources, use and exposure routes of these nanoparticles and summarize the current literature findings on their *in vivo* and *in vitro* genotoxic and carcinogenic effects. Due to the increasing evidence of their role in carcinogenicity, we have also included studies that have reported epigenetic factors, such as abnormal apoptosis, enhanced oxidative stress and pro-inflammatory effects involving these nanoparticles.

## Contents

1. Introduction
2. Genotoxicity and carcinogenicity of cobalt-based nano-particles
3. Genotoxicity and carcinogenicity of nickel-based nano-particles
4. Genotoxicity and carcinogenicity of copper-based nano-particles
5. Conclusion

## 1. Introduction

In the new millennium, nanotechnology has broadened the horizon for innovators, producers and consumers in almost all sectors, by enabling the engineering of functional systems at the molecular level. Currently, materials derived from this technology are used in catalysis and electronics, two-dimensional nanotubes and nanowires for optical and magnetic systems, and as nanoparticles used in cosmetics, pharmaceuticals and coatings (1). Nanoparticles are particles or objects with at least one of their three dimensions in the range of 1–100 nm (2,3). Nanoparticles have existed in our natural environments (volcanic ash, ocean spray, magnetotactic bacteria and mineral composites) before engineered nanoparticles were produced or were unintentionally produced (by-products of industrial processes) (4). Engineered nanomaterials, including nanoparticles and nanofibers, are generally classified into carbon-based materials, metal-based materials, dendrimers and composites (5,6). Although humans and the environment have been able to tolerate, avoid or adapt to naturally occurring nanoparticles, what is of relevance now is the intentional and unintentional production of nanoparticles and their accumulation that pose a potential environmental and health risk.

The quantum properties these nanoscale particles possess make them unpredictable and, thus, may cause toxicity, which may lead to genotoxicity and carcinogenicity. The key factor in nanoparticle toxicity is their minute size, apart from the chemical composition, shape and particle aging. Often smaller than cellular organelles and cells, this allows the penetration of basic biological structures, which may in turn disrupt their normal function (7,8). In addition, the physical and chemical properties of a nanoparticle cannot be simply predicted from the properties of a fine particle with the same chemical composition. This is supported by studies which have shown that nanoparticles are more toxic than the corresponding fine particles (9–14).

The possible toxic effects that are caused by nanoparticles include tissue inflammation and altered cellular redox balance toward oxidation, which causes abnormal cell function or cell death. Oxidative stress is a normal cellular process involved in numerous aspects of cellular signaling (15–17). Oxidative stress results as a consequence of an imbalance between the production and manifestation of reactive oxygen species (ROS), and the ability of biological systems to readily detoxify the reactive intermediates or to repair the resulting damage. Elevated cellular oxidative stress has been noted by numerous studies following exposure to nanoparticles of different metals (11,18–24). In addition, the intracellular response to nanoparticles, degree of cytotoxicity and potential mechanism of toxicity of cells to nanoparticles is also dependent on the target cell type (25). These cells include those that are located at the most likely points of entry for nanoparticles, such as the lungs, skin and gastrointestinal tract (GIT). Other possible routes of exposure, as in the field of medicine, are through injection and implantation (26,27).

Recently, numerous studies have evaluated the potential for genotoxicity and carcinogenicity of metal-based nanoparticles, particularly cobalt-, nickel- and copper-based nanoparticles. This review focuses on current knowledge on the potential for genotoxicity and carcinogenicity of cobalt-, nickel- and copper-based nanoparticles used in *in vitro* and *in vivo* mammalian studies. In addition, studies that have indicated epigenetic factors, such as enhanced oxidative stress, pro-inflammatory effects and abnormal apoptosis in their results are also considered.

## 2. Genotoxicity and carcinogenicity of cobalt-based nano-particles

### 

#### Sources of cobalt

Naturally, cobalt occurs as only one stable isotope, cobalt 59. However, through neutron activation of cobalt 59, another isotope named cobalt 60 is produced. Cobalt 60 is a commercially important radioisotope used as a radioactive tracer, in the production of γ-rays and as cold sterilization for food in certain countries. In animals, cobalt forms the center of the coenzyme cobalamin or vitamin B12, which is an essential trace element.

#### Uses of cobalt nanoparticles

As a nanoparticle, cobalt’s metallic form appears black. Cobalt-based nanoparticles may be produced as cobalt oxide, organic metal compounds or biopolymers (28). In biomedical applications, cobalt-based nanoparticles are used as starting materials for the formation of magnetic polymer microspheres and dextran coating. Colloidal cobalt nanoparticles, such as cobalt ferrite (CoFe_2_O_4_), have applications in information storage and energy (29). In medicine, cobalt has been known as a highly effective magnetic resonance imaging (MRI) contrast agent, in combination with gold (30), iron and graphite (31), and platinum (32). It has also been investigated for use in cancer therapy (32) and anaerobic waste water treatment (32–34).

#### Cobalt nanoparticle exposure

Human exposure to cobalt occurs from industry, the environment or after joint replacement from the cobalt-chrome alloy in implants. In occupational settings, exposure to cobalt and its compounds may lead to various lung diseases, including interstitial pneumonitis, fibrosis and asthma (27,35–37). The carcinogenic potential of cobalt and its compounds were evaluated by IARC in 1991, which concluded that there was inadequate evidence for carcinogenicity in humans (lung cancer) but sufficient evidence in experimental animals (36,38). In recent years, the application of cobalt nanoparticles ranges from industry to medicine, but research data on the bio-effects, particularly in comparison with their fine size particles, are limited. This is likely due to their relatively short history of production and application. In addition, it should be mentioned that little is specifically known about the toxicology of cobalt metal particles including both fine and nanoparticles, likely since it was assumed, as for other metals, that the biological activity of a metal particle, including genotoxicity and carcinogenicity, was mediated by the ionic form and could be determined by evaluating its soluble compound (36). However, evidence shows that, in contrast to what is generally hypothesized for the majority of metals, the biological activity of cobalt metal particles is not exclusively mediated by the ionic form dissolved in biological media (36).

### In vitro studies

#### Cobalt nanoparticles

Cobalt metals are known to be genotoxic *in vitro*, whereas cobalt ions are known to be carcinogenic to rodents (39). Reviews on cobalt compounds (40) and metal particles (36) have surmised that cobalt (compound or metal) particles are genotoxic. These conclusions were reached based on the effects of cobalt metals and compounds on mammalian cells *in vitro*. The genotoxic effects noted included DNA strand breaks, sister chromatid exchanges and aneuploidy, morphological transformation (40) and interference with repair processes of damaged DNA (36). These results were in part, similar to those induced by cobalt nanoparticles. According to a study on BALB/3T3 mouse fibroblast cells, it was observed that cobalt nanoparticles (>1 μM) induced an increased production of single- and double-strand DNA breaks as well as chromosomal aberrations in the form of micro-nucleate binucleate cells (41). However, this was shown to be reduced at higher concentrations (100 μM). In addition, cobalt nanoparticles induced a significant increase in the formation of type III foci (morphologically transformed colonies). Studies that used peripheral leukocytes have also shown cobalt nanoparticles to be genotoxic, when compared with cobalt ions (Co^2+^) in a dose-dependent manner. Genotoxicity was shown as increases in the % tail DNA shown by the comet assay (42) and positive results of micro-nulceate binucleate cells provided by the micronucleus test (42). In these studies, the particle size ranged from 100 to 500 nm. The increase in pseudotumors due to prosthetic implants led Kwon *et al* (43) to investigate the cause. They demonstrated that at a particle size of 30–35 nm, cobalt nanoparticles showed cytotoxicity in macrophages *in vitro* at a concentration of 1×10^12^ particles/ml. They postulated that the high concentration of cobalt required for toxicity of macrophages *in vitro* meant that there was an increased production of cobalt nanoparticles *in vivo*. Thus, ingestion of the nanoparticles by macrophages produces pseudo-tumors at implant sites. Marked differences on pro-inflammatory response and oxidative stress by cobalt nanoparticles in human endothelial cells *in vitro* were observed in another study (44).

These findings suggest that nano-sized cobalt particles are internalized by human leukocytes and interact with DNA, leading to the observed genotoxic effects. Therefore, including fibroblast cells, it can be postulated that cobalt nanoparticles have a genotoxic effect on the reticuloendothelial system.

#### Cobalt-chrome nanoparticles

Numerous other studies used the cobalt-chrome alloy nanoparticle to conduct their research, in the majority of these studies the fibroblast cell was predominantly used (45–48), and almost all observed cobalt-chrome nanoparticles to be cytotoxic and genotoxic to this cell line. For example, a study comparing fine and nanoparticle alloys of cobalt-chrome on human fibroblast cells at equivalent volumetric doses noted that the nanoparticles generated free radicals, cell DNA damage, cytotoxicity and aneuploidy (45). A complex form of aneuploidy was also reported by Figgitt *et al* (46) who conducted similar studies on human fibroblast cells that were derived from the peripheral blood of individuals exposed to cobalt-chrome and cobalt (II). Other studies have reported that cobalt-chrome increased the production of micro-nucleate binucleate cells (47), caused chromosomal losses, gains and deletions (47), tetraploidy (49) and DNA double-strand breaks (49). In a study conducted by Bhabra *et al* (48), it was reported that cobalt-chrome nanoparticles (29.5 nm) damage human fibroblast cellular DNA across an intact cellular barrier without crossing the barrier. They suggested that the damage is mediated by a novel mechanism involving transmission of purine nucleotides (such as ATP) and intercellular signaling within the barrier through connexin gap junctions or hemi-channels and pannexin channels.

An elevated cellular inflammatory response has been noted in numerous studies following exposure to nanoparticles (50–54), including cobalt nanoparticles. Guildford *et al* (55) investigated the effect of various types of nanoparticles on key components of the host response, such as clot formation and inflammatory cells. The results showed that cobalt nanoparticles (28 nm) stimulated cells to acquire a macrophage phenotype able to secrete higher levels of the pro-inflammatory cytokine, tumor necrosis factor α (TNFα). A large variety of soluble factors, including cytokines [e.g. interleukins (IL)] and tumor necrosis factor (TNF) protein families, migration inhibition factors, ROS and reactive nitrogen species, are inflammatory response factors which mediate inflammation. Under normal physiological conditions, these factors are important protective defenses against tissue injury or infection. However, they are also capable of promoting DNA damage, such as chromosomal fragmentation, DNA point mutations, inhibition of DNA repair and formation of methylation patterns that may lead to altered gene expression profile and the formation of DNA adducts (56). In addition, recent research evidence revealed that the inflammatory microenvironment in and around tumors is an indispensable participant in the neoplastic process (57).

The majority of studies conducted on cobalt-chrome alloys mentioned presently indicate nanoparticles of cobalt-chrome alloys to be genotoxic. The genotoxicity was either via genetic factors, such as chromosomal aberrations and DNA damage in the form of single- and double-strand breaks, or via transmission of nucleotides through gap junctions. They may even cause genotoxicity through epigenetic factors, such as inflammation.

#### Cobalt-oxide nanoparticles

Engineered cobalt oxide (Co_3_O_4_) nanoparticles were shown to enter cells rapidly and remain confined to vesicles, thus causing a rapid induction of ROS in human cell lines (58).

#### Tungsten carbide-cobalt (WC-Co) nanoparticles

In an *in vitro* study (59), when tested over a range of cobalt equivalent concentrations (1.5–15 μg/ml), WC-Co particles were shown to cause significantly more DNA breaks than cobalt metal particles alone, both on isolated human DNA and in cultured human lymphocytes (alkaline elution and comet assays). In addition, this DNA damage could be inhibited by scavenging activated oxygen species. In another study, WC-Co nanoparticles were demonstrated to induce a higher level of oxidative stress and activated the activator protein-1 (AP-1) and nuclear factor-κB (NF-κB) more efficiently in JB6^+/+^ cells than WC-Co fine particles (60). It also had a high potency to stimulate mitogen-activated protein kinases (MAPKs), including extracellular signal regulated kinases (ERKs), P38 and c-Jun N-terminal kinases (JNKs). In human keratinocyte cells (HaCaT) at concentrations of 3 μg/ml for an exposure of 3 h and 3 days, WC-Co nanoparticles were able to exert responses similar to those of free cobalt ions (CoCl_2_), particularly the induction of hypoxia-like effects via interactions with HIF-1α in human keratinocytes (61). In a study performed on WC-Co nanoparticles, it was concluded that ROS may act as a major contributor in nano WC-Co particle-induced adverse health effects (62).

WC-Co nanoparticles cause genotoxicity via DNA damage, oxidative stress, activation of activator proteins and proteins in the mitogenic pathways. These were confirmed by studies which compared fine and nanoparticles of WC-Co.

#### Cobalt-ferrite nanoparticles

Cobalt-ferrite nanoparticles (CoFe_2_O_4_; 6–12 nm) were used to investigate the interaction with nucleic acid (63). The investigators in this study observed that the research data collectively revealed that there was an interaction between CoFe_2_O_4_ nanoparticles and nucleic acid. The investigators presumed that the linkage was based on a coordination interaction of the phosphate groups and the oxygen atoms on the heterocyclic bases of DNA with metal ions on the particle surface.

The *in vitro* studies demonstrated that cobalt nanoparticles induced DNA strand breaks, micronuclei formation, chromosomal aberrations (aneuploidy, polyploidy and tetraploidy) and morphological transformation of mammalian cell lines. Cobalt nanoparticles exhibited higher genotoxicity than cobalt fine particles and ions. Cobalt nanoparticles were also shown to cause inflammation and oxidative stress. They are also proven to have toxic effects towards both anchorage and non-anchorage cells *in vitro*, and the majority of these findings were derived from studies performed on fibroblast cells. This may be due to the increased use of a cobalt-chrome alloy in replacement surgeries.

### In vivo studies

Only one *in vivo* study was retrieved for the evaluation of the carcinogenesis of cobalt nanoparticles. Hansen *et al* (64) implanted cobalt fine particles and nanoparticles (50 and 200 nm) bilaterally (i.e. subcutaneously with fine particles, and intramuscularly with nanoparticles for each animal) at the vertebral column of rats to investigate the carcinogenesis of cobalt particles. In five out of six implanted rats, the sites of nanoparticle implantation developed nodules. Morphological and histochemical biomarker investigations revealed that these nodules were malignant mesenchymal tumors. On the contrary, malignant mesenchymal tumors were not observed around the fine particles. In the subcutaneous area of fine particle implantation, discrete fibrosis and discrete inflammatory infiltrate were observed, but not granulomas. A model of the neoplasia sequence for the carcinogenesis of cobalt nanoparticles was summarized as follows: inflammation→preneoplasia→neoplasia. This difference in carcinogenic potency suggests the need to develop a separate risk estimate for cobalt fine and nanoparticles, and to develop separate recommendations for occupational and environmental exposures to each size range. However, there is also a need for further *in vivo* animal studies and epidemiological investigations.

The experimental evidence indicates that both cobalt fine and nanoparticles exert certain genotoxic and carcinogenic activity in *in vitro* and *in vivo* experimental systems. In addition, one *in vivo* study in rats demonstrated that cobalt nanoparticles induced malignant mesenchymal tumors, whereas cobalt fine particles did not at the equivalent treatment dose. Since there is evidence of genotoxicity in the studies that have utilized fibroblast cells, *in vivo* animal studies and epidemiological investigations that focus on replacements or implants that are cobalt-based are recommended.

## 3. Genotoxicity and carcinogenicity of nickel-based nano-particles

### Sources of nickel

Nickel is the fifth most abundant element in the world. Approximately 85% of nickel is used in combination with other metals to make alloys, the best known of which is stainless steel.

### Uses of nickel nanoparticles

Alloys of nickel are used in the home, architecture, health care, food processing and throughout industry. Non-alloys of nickel are used in nickel plating and chemical applications, including in rechargeable batteries, electronics, power tools, transport and emergency power supplies. Nickel-based nanoparticles have a wide variety of applications in industry. For example Ban *et al* (65) investigated alloys of copper and nickel at the nanometer scale for use in controlled magnetic hyperthermia applications. Metallic nickel nanoparticles have also shown potential for use as electrode materials in multilayer ceramic capacitors (MLCC) (66). Researchers have also explored the potential for nickel nanoparticles in the form of nanorings as memory cells (67). Zhao *et al* (68) conducted a comprehensive general review of nickel and nickel compounds.

### Nickel nanoparticle exposure

Lung inhalation is the major route of nickel exposure. However, it may also be ingested or absorbed through the skin. The primary target organs are the lungs and kidneys (69). According to Kasprzak *et al* (70) ‘the most adverse effects of exposure to nickel are skin allergies, lung fibrosis, and lung cancer’. Some of the common nickel compounds are nickel oxide (NiO), nickel chloride (NiCl_2_) and nickel sulphide (Ni_2_SO_4_). Nickel compounds are classified by IARC as group 1, carcinogenic to humans. Whereas, metallic nickel is classed as group 2B, possibly carcinogenic to humans. In 2008, nickel was voted as the allergen of the year by the American Contact Dermatitis Society following an article by Kornick and Zug (71) on nickel. Numerous experimental and epidemiological studies, as well as reviews, have shown metallic nickel and nickel compounds to be carcinogenic (72–77).

Nickel-based nanoparticles are new products and have been widely used in industry in recent years (78,79). Their characteristics include a high level of surface energy, high magnetism, low melting point, high surface area and low burning point. However, concerns have been expressed that these same properties of nickel-based nanoparticles may present unique bioactivity and challenges to human health (80). Although little is known about the effects of particle size relative to speciation, it is worth mentioning that the size of the nickel-based nanoparticles may play an important role in the biological effects (81).

### In vitro studies

#### Nickel nanoparticles

Numerous studies have examined the genotoxicity of nickel compounds by using various toxicological test systems in the past 30 years (68). The genotoxicity of metallic nickel fine and nanoparticles has not been demonstrated yet, except for the indications of a few studies. When compared to the known genotoxic compound titanium oxide, alveolar epithelial (A549) cells exposed to nickel nanoparticles (100 nm) caused greater apoptotic damage in both flow cytometry and DNA fragmentation studies (82). The extent of DNA fragmentation was increased by 20–24%. The investigators in this study suggested that these effects were attributable to ROS generation. Apoptotic DNA fragmentation is a key feature of apoptosis, where DNA is cleaved into internucleosomal fragments of 180 bp (83), in response to a variety of apoptotic stimuli in a diverse range of cells. Studies performed on other nanoparticles have summarized that oxidative stress may be a key route in inducing the cytotoxicity of nanoparticles according to their findings (18,84,85). Apoptosis was also observed in the mouse epithelial (JB6) cell line by Zhao *et al* (86). It was observed that metallic nickel nanoparticles (92.32 nm) caused higher cytotoxicity and apoptotic induction than fine particles (3.34 μm) after a 24-h exposure of JB6 cells to 0.1–20 μg/cm^2^ of nickel nano or fine particles. They concluded that the Bcl-2 and Akt (used as endpoints) may play a role in preventing the release of cytochrome *c* from the mitochondria into the cytoplasm. ROS cause cell death via necrotic or apoptotic pathways. The mechanisms of cell death via ROS generation include receptor activation, caspase activation, Bcl-2 family proteins and mitochondrial dysfunction (17,87). These findings were similar to studies conducted on leukemia cells (K562 cells) where it was revealed that the nickel nanoparticles capped with positively charged tetraheptylammonium were cytotoxic to these cells at high concentrations, and subsequently induced both apoptosis and necrosis of target cancer cells (88). This demonstrated that functionalized nickel nanoparticles with positively charged groups could enhance the permeability of the cell membrane and facilitate the cellular uptake of external target molecules into leukemia K562 cells. In another study nickel nanoparticles caused a rapid and prolonged activation of the hypoxia inducible factor-1α (HIF-1α) pathway, which was stronger than that induced by soluble nickel (II) (89). They concluded that moderate cytotoxicity and sustained activation of the HIF-1α pathway by metallic nickel nanoparticles could promote cell transformation and tumor progression. The characteristics of this toxicity pathway are similar to those activated by carcinogenic nickel compounds.

Ahamed (90) showed that nickel nanoparticles induced ROS production in a dose- and time-dependent manner in A549 cells treated with 0, 1, 2, 5, 10 and 25 μg/ml nanoparticles for 24–48 h. This was indicated by a depletion of GSH and induction of ROS and lipid peroxidation (LPO). They also showed that nickel nanoparticles reduced mitochondrial function and induced the leakage of lactate dehydrogenase (LDH) in a dose- and time-dependent manner. Nickel nanoparticles (62 nm) were capable of promoting the polymerization of fibrin and the aggregation and fragmentation of platelets, leading to a moderately activated monocyte phenotype (55). Marked differences in oxidative stress and pro-inflammatory responses by nickel nanoparticles in human endothelial cells *in vitro* were also observed in another study (44).

Nickel nanoparticle genotoxicity was shown by increased DNA fragmentation that led to apoptosis. Nickel nanoparticles also caused cell death by generating ROS either by caspase activation, activation of the Bcl-2 family, activation of HIF-1α or mitochondrial dysfunction. Oxidative stress and pro-inflammatory response were also noted.

#### Nickel-oxide (NiO) nanoparticles

NiO (20 nm) was observed to increase the gene expression of heme oxygenase-1 (HO-1) and surfactant protein-D (SP-D) in A549 cells (91). The researchers of this study highlighted that an increase in gene expression of stress responsive enzymes, such as HO-1 and SP-D, and translocations of the transcription factor HIF-1α were caused by NiO nanoparticles. Pietruska *et al* (89) provided further evidence on this point. They performed physicochemical characterization of NiO and metallic nickel particles and ion bioavailability and toxicological properties in human lung epithelial cells (H460). Their results showed that NiO nanoparticles induced stabilization and nuclear translocation of the HIF-1α transcription factor followed by upregulation of its target gene, N-myc downstream regulated gene 1/Cap 43 [NDRG1(cap43)]. In this study, cytotoxicity to H460 cells was observed to occur concomitantly with activation of an apoptotic response as determined by dose- and time-dependent cleavage of caspases and PARP.

The level of intracellular ROS was also observed to increase with increasing exposure to nickel oxide nanoparticles (20 nm) on A549 cells (91). In a study investigating the inflammation potency of nickel oxide (92), well-characterized nanoparticles of nickel oxide were instilled into the lung of rats using two time points (24 h and 4 weeks) to evaluate the acute and chronic effects. The results showed that along with cesium oxide and zinc oxide, nickel and copper oxides at 10–20 nm and <50 nm respectively, had acute and chronic inflammogenic effects on the lung. Acutely, patterns of the lung showed that neutrophil and eosinophil infiltrates differed following instillation. Chronically, the nanoparticles caused neutrophilic, neutrophilic/lymphocytic, eosinophilic/fibrotic/granulomatous and fibrotic granulomatous inflammation.

The aforementioned studies have shown that both Ni and NiO appear to activate the HIF-1α pathway, which may promote cell transformation and tumor progression. Apoptosis is also a key finding in the Ni nanoparticle studies.

### In vivo studies

In a case study reported by Iannitti *et al* (93), nickel nanoparticles, including nanoparticles of other heavy metals, were indicated as the causative agents in Hodgkin’s lymphoma. Nickel nanoparticles were identified in the bone marrow biopsy and the right inguinal lymph node specimens using field emission gun-environmental scanning electron microscopy (FEG-ESEM) coupled with energy dispersive spectroscopy (EDS). This indicated the presence of heavy metal nanoparticles in cells and their involvement in the onset of Hodgkin’s disease. Evidence exists of systemic and pulmonary pathology in a human following exposure to nickel nanoparticles (94), but whether it causes carcinogenicity in animal models is a question that is still being widely investigated. The majority of *in vivo* studies on nickel nanoparticles have been focused on pulmonary pathology. Gillespie *et al* (95) used occupationally relevant dose ranges of nickel hydroxide and C57BL/6 mice as their animal models. These forms of nickel-based nanoparticles were used since they are highly favorable for use as a chemical energy source in power or energy markets. Their studies showed that nickel hydroxide nanoparticles are capable of inducing inflammatory effects in the lungs after both short- and long-term exposure periods. Although short-term exposure may cause reversible genetic damage, long-term persistent exposure is to be carefully considered. Long-term exposure renders the cell vulnerable to DNA aberrations that consequently lead to mutagenesis. Rats that were intramuscularly implanted with metallic nickel nanoparticles developed rhabdomyosarcoma in a study (64). However, this study showed that both nickel fine and nanoparticles caused the development of rhabdomyosarcomas. Another study that intratracheally instilled rats with 0.2 μg of nickel oxide dispersed in distilled water which had a mass median diameter in water of 26 nm showed that the expression of macrophage inflammatory protein-1α (MIP-1α) was continually increased in lung tissue and broncho-alveolar lavage fluid (BALF), whereas interleukin-1α (IL-1α) and IL-1β were increased in lung tissue and monocyte chemotactic protein-1 (MCP-1) showed a transient increase in BALF (96). This study examined the induction of 21 cytokines, including inflammation-, fibrosis- and allergy-related, by well-dispersed nickel oxide nanoparticles in lung disorders. It was concluded that overall agglomerates of nickel oxide nanoparticles have a persistent inflammatory effect and that the increase in cytokine expression and persistent increase in CC chemokine (β-chemokine) were involved in the persistent pulmonary inflammation. Two different studies by Morimoto *et al* (97) and Nishi *et al* (98) obtained similar results in studies conducted on the toxicity of nickel oxide nanoparticle and agglomerates following intratracheal instillation in male Wistar rats. Both Morimoto *et al* (97) and Nishi *et al* (98) exposed rats to nickel oxide nanoparticles (3.3 mg/kg and 26 nm mass median diameter), and were dissected at 3 days, 1 week, and 1, 3 and 6 months. In both studies, through quantitative measurement of protein by ELISA, the level of cytokine-induced neutrophil chemoattractant (CINC)-2αβ was elevated (3 days to 6 months and 3 days to 3 months). However, in the study by Nishi *et al* (98), it was observed that the level of CINC-1 was increased from 3 days to 3 months, and CINC-3 was increased at 3 days, but subsequently decreased. They also observed the infiltration of neutrophils and alveolar macrophages in lung tissue. BALF cell count was also increased consistently, with a significant increase in neutrophil and alveolar macrophage count in both studies. In the study by Morimoto *et al* (97), it was concluded that nanoparticle agglomerates of nickel oxide induced a persistent inflammatory response, while Nishi *et al* (98) suggested that CINC was involved in lung injury from nickel oxide nanoparticles. Nickel hydroxide nanoparticles demonstrated stronger inflammogenic potential then the other nanoparticles (99). This study was performed to examine ROS and inflammatory responses in mice exposed to each type of nanoparticle for 4 h in a whole-body inhalation system. Lipid peroxide levels were increased 24 h after instillation, but decreased 3 days later in another study where nickel oxide nanoparticles were intratracheally instilled in rats (91).

The *in vivo* investigations performed with nickel nanoparticles show that nickel nanoparticles cause cancers. They also induce other cellular and molecular effects that have the potential to be carcinogenic, such as inflammation and induction of oxygen radicals. The case studies also show that nickel nanoparticles caused pulmonary pathology similar to those in rats and together with other heavy metal nanoparticles, may cause Hodgkin’s lymphoma.

The limited *in vitro* experimental results show that the nickel nanoparticles induced DNA fragmentation and activation of the HIF-1α pathway in cultured cells. The nanoparticles also induce persistent inflammation in *in vivo* rat models explained by increases in inflammatory markers. One *in vivo* experimental study in rats demonstrated that both nickel nano and fine particles caused the formation of rhabdomyosarcomas and one retrospective case study showed involvement of nickel nanoparticles in Hodgkin’s lymphoma.

## 4. Genotoxicity and carcinogenicity of copper-based nano-particles

### Sources of copper

Copper in its pure state is rarely found in nature, but it is found combined with other chemicals in an ore. Worldwide, there are approximately 15 copper ore mines in 40 countries. According to a British Geological Survey by Brown *et al* (100), 15,800,000 tons of copper were produced in the world between 2005 and 2009. Copper is also an essential micronutrient which is necessary for the proper growth, development and maintenance of bone, connective tissue, brain, heart and numerous other organs (101–103). Copper is also involved in the stimulation of the immune system to fight infections, repair injured tissues and promote healing (104), and it also aids neutralization of ‘free-radicals’, which cause severe cell injury (105). The average level of stored copper in the body (mostly in the liver) is approximately 120–150 mg. Copper may be absorbed by the stomach, but the majority is absorbed by the small intestine. In the blood, it is observed bound to proteins. Under normal physiological conditions, copper is mostly excreted via bile that is released into the GIT with minimal copper reabsorbed by intestinal cells. This allows copper to be conserved and tightly regulated. Copper, therefore, is useful in both the physical and the biological aspects of humans.

### Uses of copper

Copper is commonly used in the production of electrical wire, household kitchen appliances, pipes and tubes, automobile radiators and as a pigment and preservative for paper, paint, textiles and wood. Copper nanoparticles are used as additives in lubricants, polymers or plastics, metallic coatings and ink (106,107), they are also used as bioactive coatings that are capable of inhibiting target microorganisms such as *Escherichia coli* and *Staphylococcus aureus* (108). Copper nanoparticles are also developed for temperature and pressure sensing (109), and as hydrogen catalysts in fuel cells. They are also investigated for use in the design of bioactive nanocomposites, such as biomedical silicones, to give it strong nanoparticle properties (110). Both copper fine and nanoparticles have a wide variety of industrial and commercial uses and are still being explored. This is particularly true for copper nanoparticles.

### Copper nanoparticle exposure

Occupational exposure of copper dusts or fumes is harmful to human health, including an increased risk of cancer among copper smelter workers (111). In the early studies on the genotoxicity and carcinogenicity of water soluble copper compounds, such as copper sulfate, they were observed to be genotoxic, with characteristics including the induction of chromosomal aberrations and micronuclei in White Leghorn chick bone marrow cells (112) and chromosomal aberrations in Swiss mice (113). However, the data on the genotoxicity and carcinogenicity of water insoluble copper particles are scarce.

### In vitro studies

#### Copper nanoparticles

Copper nanoparticles have been demonstrated to be extremely reactive in a simulative intracorporeal environment (114). The reason for this high reactivity was due to copper nanoparticles (23.5 nm) consuming hydrogen ions in the stomach at a faster rate, which then are converted further into cupric ions whose toxicity is known to be high *in vivo*. Metal ions are known to have a high affinity for electron-rich molecules such as DNA, but studies have shown that copper nanoparticles are able to interact with DNA. This was demonstrated by a study in which copper nanoparticles (4–5 nm) caused a dose-dependent degradation of isolated DNA molecules via the generation of singlet oxygen (^1^O_2_) in 937 and HeLa cells (115). Singlet oxygen is the active species in photodynamic therapy for cancer (116).

#### Copper-oxide nanoparticles

Since there is a published review paper available with regard to the effects of DNA damage induced by copper oxide nanoparticles in *in vitro* studies (117), only a few are mentioned here. DNA damage as a result of oxidative stress, identified by increased levels of 8-isoprostane and the ratio of glutathione disulfide (GSSG) to total glutathione in human airway epithelial (Hep-2) cells has been reported (18). Oxidative stress increased the expression of plasminogen activator inhibitor-1 (PAI-1), by mediating p38 phosphorylation in endothelial cells treated with copper oxide nanoparticles (42 and 200 nm) (118). Elevated oxidative stress may lead to DNA damage, which in turn has the potential for carcinogenesis. In another study on A549 cells, copper oxide nanoparticles were the most potent with regard to cytotoxicity and DNA damage (119). The toxicity may have been caused by copper ions released in the cell medium. It was also observed that CuZnFe_2_O_4_ particles were rather potent in inducing DNA. Copper nanoparticles (<100 nm) were also shown to be more toxic to human A549 cells than copper fine particles (120) and were also reported to induce toxicity of sensory neurons (121). The latter study examined the concentration (10–100 μM) and size-dependent (40, 60 and 80 nm) effects of copper nanoparticles on the survival of dorsal root ganglion (DRG) neurons of rats in cell culture for 24 h. The DRG neurons showed the presence of vacuoles and detachment of certain neurons from the substratum, they also exhibited disrupted neurite network in those exposed to copper nanoparticles. All the sizes tested had a significant toxic effect on DRG neurons compared to the controls. Copper was also shown to be intracellularly deposited by rubeanic acid staining.

The copper nanoparticles and their compounds caused a range of effects, including oxidative stress, cytotoxicity, neurotoxicity, DNA damage and DNA lesions in a variety of cell lines.

### In vivo studies

According to the Hodge and Sterner scale, copper nanoparticle toxicity is class 3, moderately toxic. Chen *et al* (106), demonstrated that copper nanoparticles particularly target the liver, kidneys and spleen in experimental mice. At a particle size of 25 nm and dosage range of 108–1080 mg/kg, copper nanoparticles caused atrophy of the spleen and discoloration of both the spleen and kidneys. Upon histopathological examination the kidneys exhibited glomerulonephritis. Alveolitis, perivasculitis and a significantly high lavage of cytokines were also observed. Cho *et al* (92) instilled well-characterized copper oxide nanoparticles into the lung of rats to evaluate their inflammatory potency. Two time points were used (24 h and 4 weeks) to evaluate the acute and chronic effects of these particles. Their results showed that copper oxide nanoparticles at 10–20 and <50 nm, respectively, had acute and chronic inflammogenic effects on the lung. Acutely, patterns of the lung showed neutrophil and eosinophil infiltrates that differed following instillation. Chronically, the nanoparticles caused neutrophilic, neutrophilic/lymphocytic, eosinophilic/fibrotic/granulomatous and fibrotic granulomatous inflammation.

Persistence of inflammation markers 3 weeks post-exposure was also observed by other investigators (122), who studied whole-body inhalation exposures performed on mice at concentrations of 3.6 mg/m^3^. Unresolved inflammation may lead to DNA aberrations that may be mutagenic. Yang *et al* (123) used male Wistar rats to investigate the mechanisms of copper nanoparticle-induced hepatotoxicity through identification from hepatic gene expression profiles that were phenotypically associated with conventional toxicological outcomes. Through histopathological studies of the rats administered with differing concentrations of copper nanoparticles and micro-copper (size unavailable), the liver exhibited scattered, punctate hepatocytic necrosis in all rats in the high-dose group. After functionally categorizing identified genes from the high-dose group, their results showed that genes related to oxidoreductase activity, metabolism and signal transduction were involved in the development of the phenotypes. Their study also demonstrated that there was an increase in aspartate transaminase, triglycerides, total bilirubin, total bile acid levels and a decrease in body weight. Punctate necrosis of hepatocytes in the liver was observed by another study which used nasal instillation of 23.5-nm copper nanoparticles in mice 3 times/week (124). They also observed swelling in the renal glomerulus and severe lesions associated with the decreased number of olfactory cells, and dilapidated laminated structures were also observed in the olfactory bulb. These were observed in the high-dose group (40 mg/kg body weight), but not in the low-dose group (1 mg/kg body weight). Sharma *et al* (125) examined neurotoxicity and neuropathology caused by silver, aluminum and copper nanoparticles that were approximately 50–60 nm in rats and mice. These were injected by intraperitoneal (50 mg/kg body weight), intraveneous (30 mg/kg body weight), intracarotid (2.5 mg/kg body weight) or intracerebroventricular administration (20 μg to both mice and rats) at 24 h after administration. Alterations in the blood-brain barrier were observed in several regions of the brain and spinal cord through Evans blue and radioiodine studies. Decreased cerebral blood flow, pronounced brain edema, neuronal cell injuries, glial cell activation, heat shock protein upregulation and loss of myelinated fibers were observed in mice exposed to silver and copper nanoparticles, particularly when administered into the systemic circulation or the brain ventricular spaces.

As indicated by these studies, copper nanoparticles induce inflammation of organs, including the lung, kidney and spleen in mice. The most profound effects were observed in liver, even when the copper nanoparticle route of administration and animal model differed among studies. The copper nanoparticles were also shown to be neurotoxic and neuropathologic. However, apart from the induction of other types of pathology, none of these studies reported carcinogenesis.

## 5. Conclusion

The number of studies on these three transitional metal-based nanoparticles is limited, although direct genotoxicity endpoints for cobalt outweigh that of nickel and copper comparatively. Well-designed studies, particularly *in vivo* studies, are required to elucidate the genotoxicity and carcinogenicity of cobalt, nickel and copper nanoparticles. Overall, changes in gene expression, apoptosis, oxidative stress and persistent inflammation were the major effects of cobalt-, nickel- and copper-based nanoparticles that may predispose to carcinogenicity. Oxidative stress also poses a significant threat since it may lead to DNA, protein and lipid damage (126). The number of *in vivo* studies performed is far fewer than the *in vitro* studies for all three elements, whether as compounds or metals. With the exception of copper nanoparticles, both cobalt and nickel nanoparticles have been shown to be carcinogenic *in vivo*.

In conclusion, both *in vitro* and *in vivo* methods are useful in studying nanoparticle toxicity and carcinogenicity, but compared to *in vivo* and *in vitro* methods are much more time- and cost-efficient. Thus, the majority of studies have been performed using *in vitro* methods that utilize various cell lines (86,127). It has now been documented that different nanoparticles elicit different responses from different cell lines or biological systems. With the exception of copper nanoparticles, cobalt and nickel nanoparticles have shown genotoxic effects and also cases of carcinogenesis in *in vivo* studies. The majority of *in vivo* studies utilize rats or mice as experimental animals. The existing studies on cobalt, nickel and copper have focused on the biological distribution of these particles following exposure through the lungs, skin and GIT.

The exact mechanisms of cobalt- and nickel-based nanoparticle-induced carcinogenesis in experimental animals are not clear. Enhanced oxidative stress, inflammatory response and abnormal apoptosis may play major roles in the carcinogenicity of cobalt- and nickel-based nanoparticle-induced carcinogenesis.

In addition, this review has shown that these metal-based nanoparticles (cobalt, nickel and copper) are particularly lacking in *in vivo* genotoxicity and carcinogenicity studies and it would be beneficial to further the knowledge of their genotoxicity and carcinogenicity. We also recommend more epidemiological studies to be performed on prosthetic implants that are cobalt-based and a clear definition of the particle size within studies.
